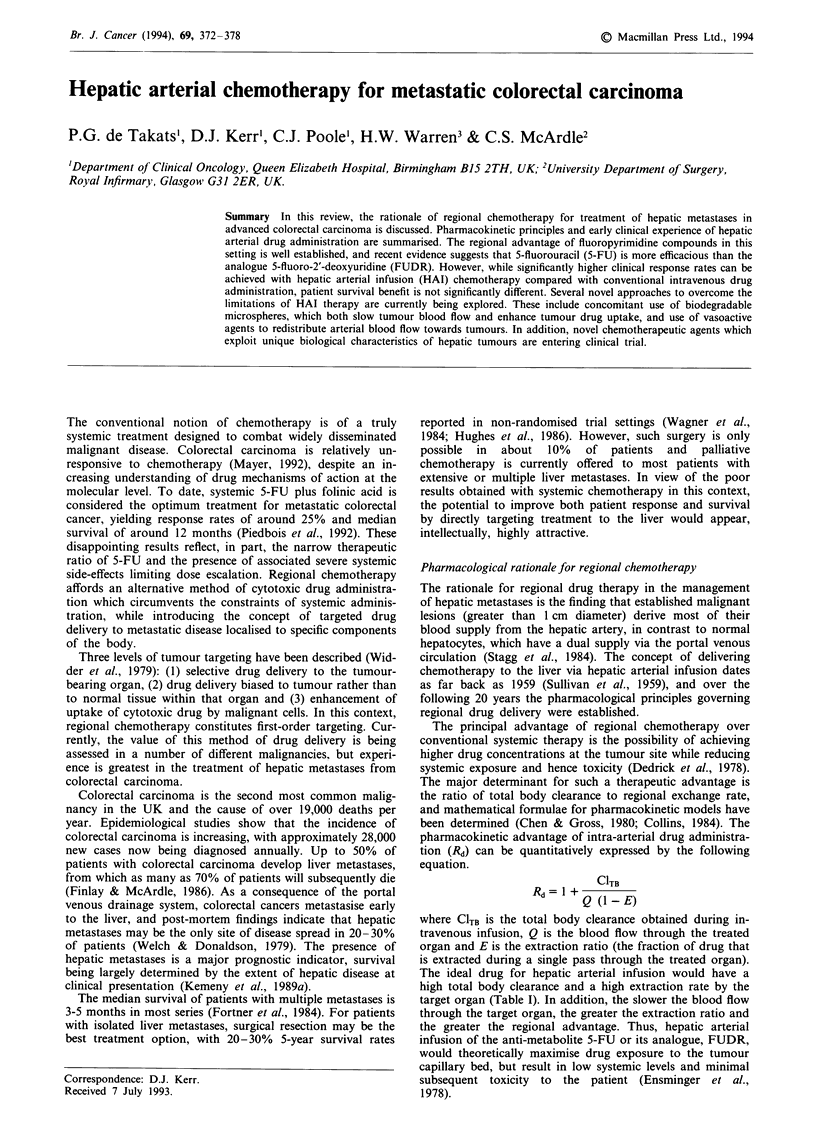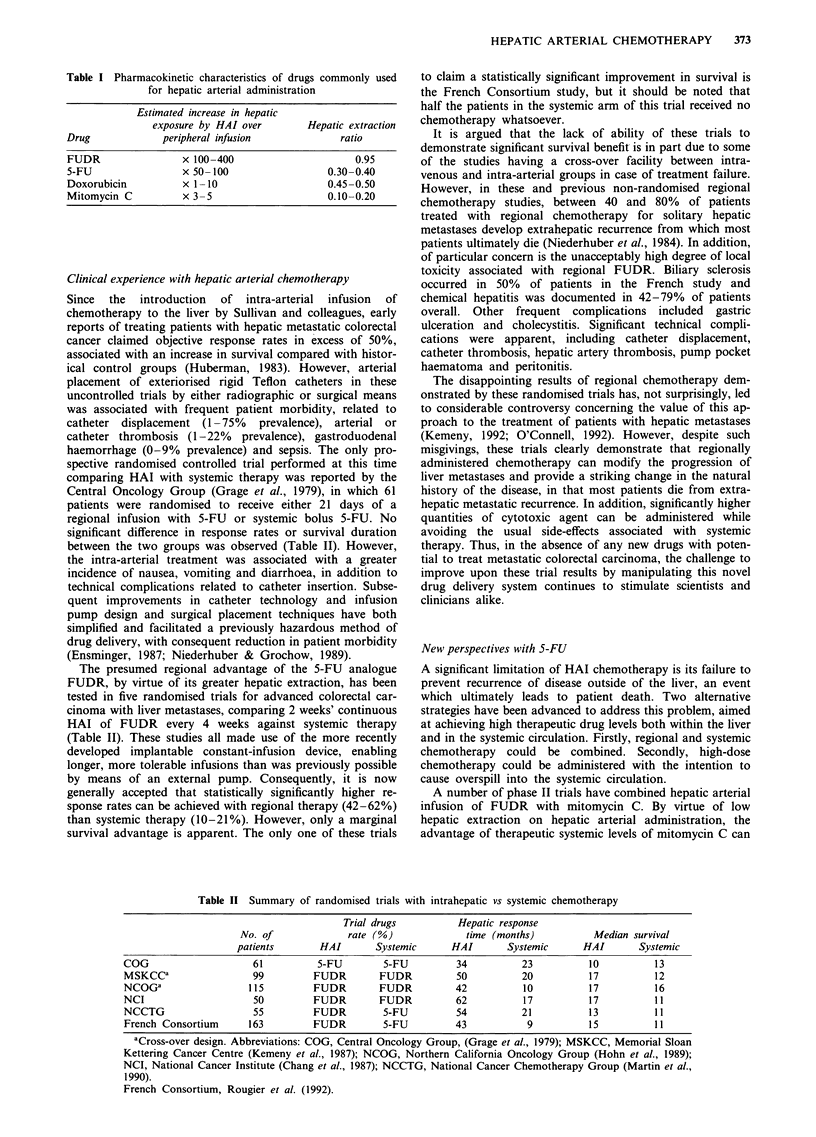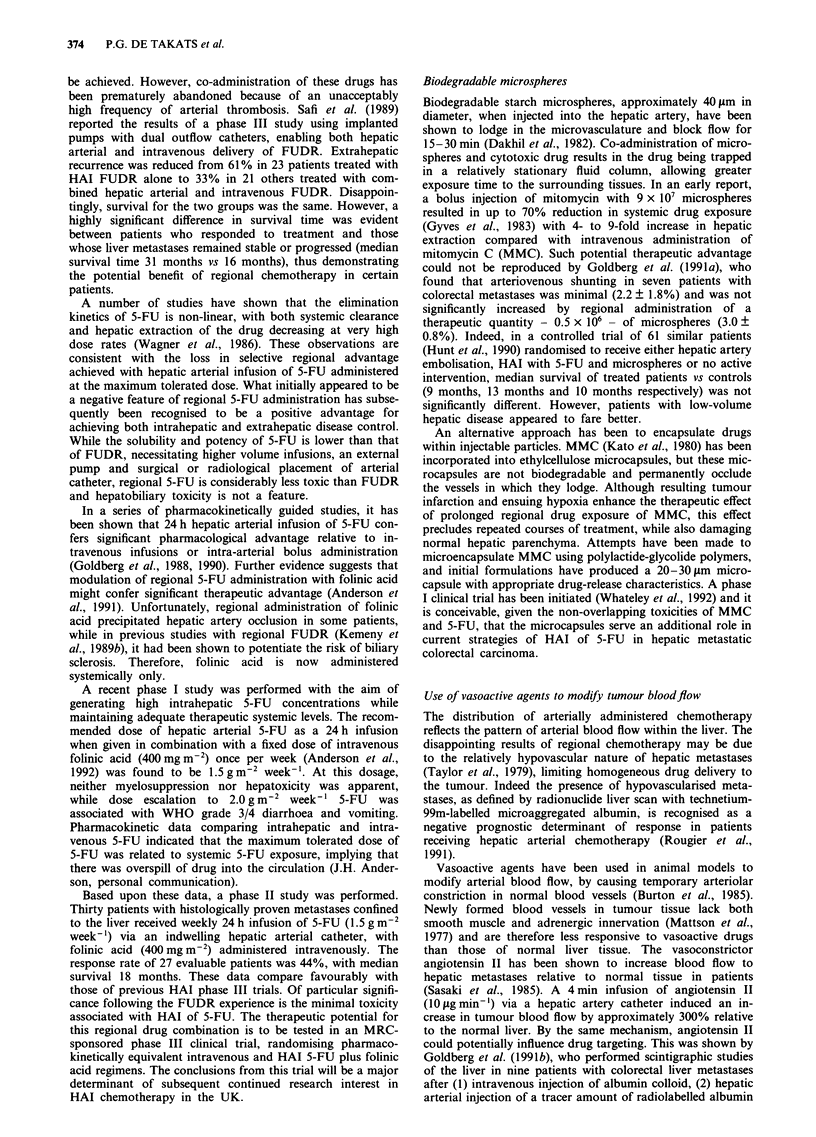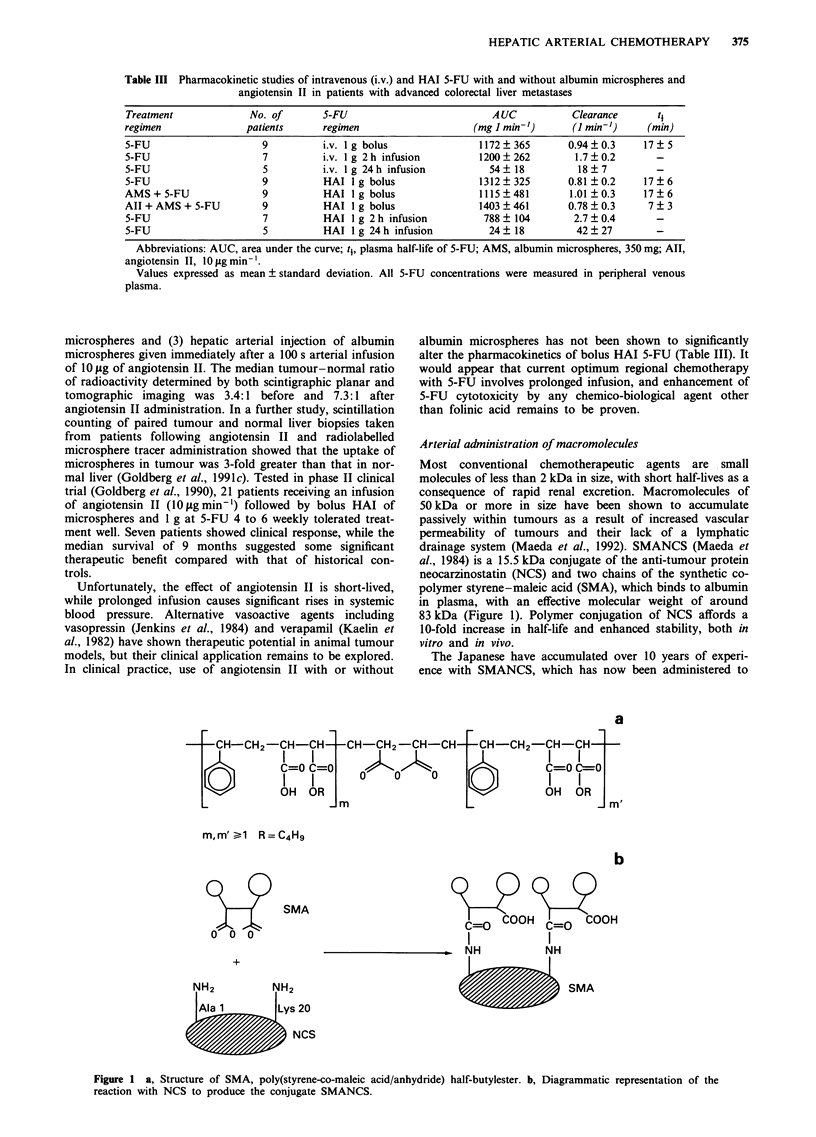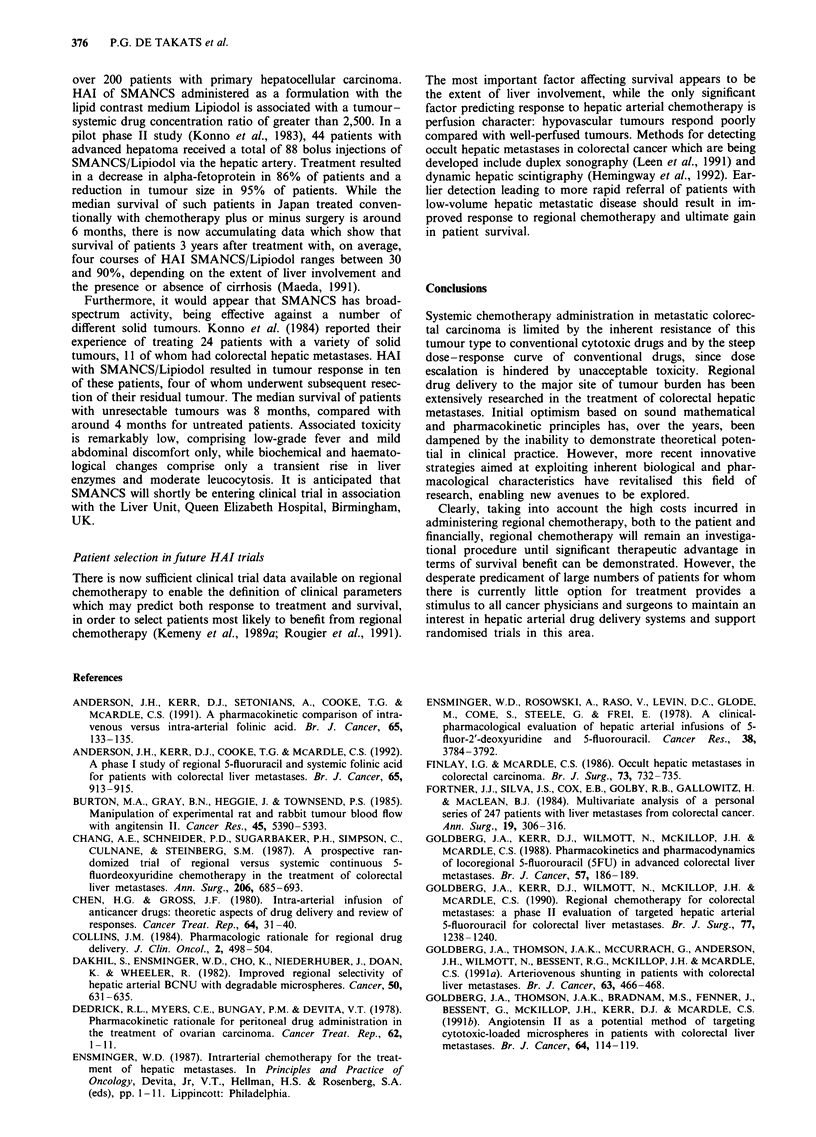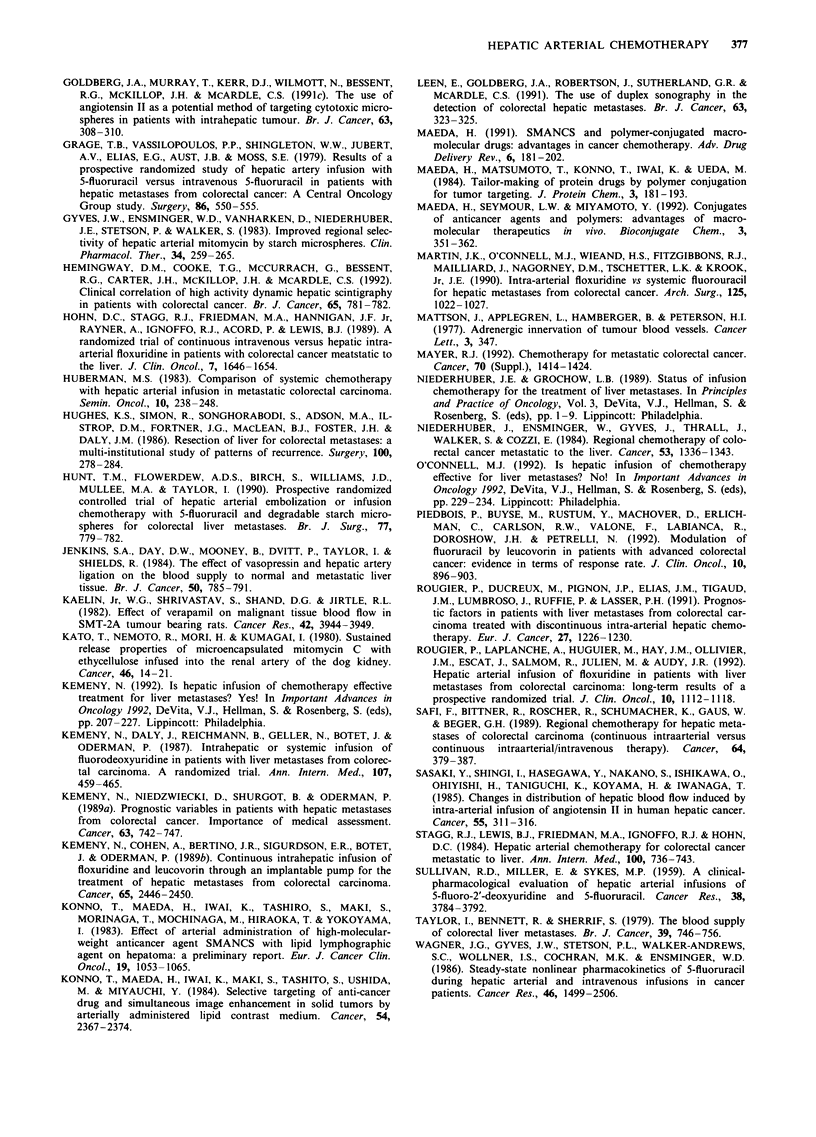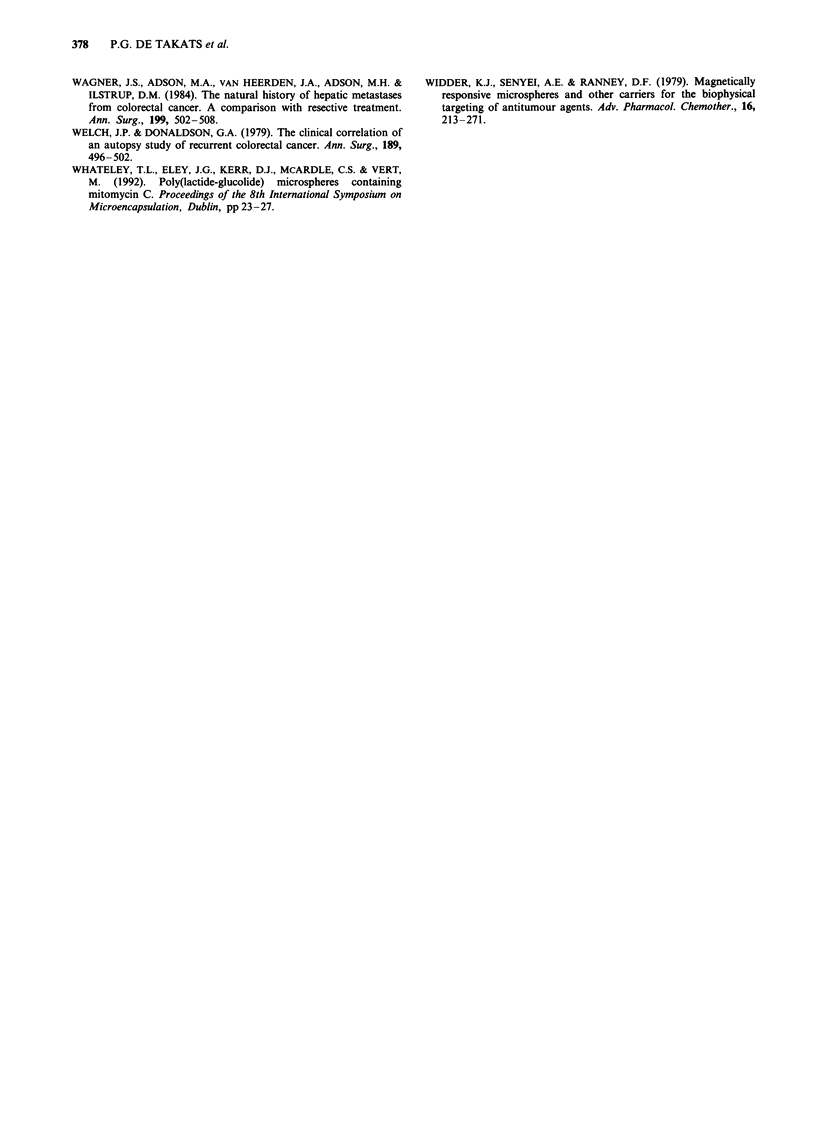# Hepatic arterial chemotherapy for metastatic colorectal carcinoma.

**DOI:** 10.1038/bjc.1994.68

**Published:** 1994-02

**Authors:** P. G. de Takats, D. J. Kerr, C. J. Poole, H. W. Warren, C. S. McArdle

**Affiliations:** Department of Clinical Oncology, Queen Elizabeth Hospital, Birmingham, UK.

## Abstract

In this review, the rationale of regional chemotherapy for treatment of hepatic metastases in advanced colorectal carcinoma is discussed. Pharmacokinetic principles and early clinical experience of hepatic arterial drug administration are summarised. The regional advantage of fluoropyrimidine compounds in this setting is well established, and recent evidence suggests that 5-fluorouracil (5-FU) is more efficacious than the analogue 5-fluoro-2'-deoxyuridine (FUDR). However, while significantly higher clinical response rates can be achieved with hepatic arterial infusion (HAI) chemotherapy compared with conventional intravenous drug administration, patient survival benefit is not significantly different. Several novel approaches to overcome the limitations of HAI therapy are currently being explored. These include concomitant use of biodegradable microspheres, which both slow tumour blood flow and enhance tumour drug uptake, and use of vasoactive agents to redistribute arterial blood flow towards tumours. In addition, novel chemotherapeutic agents which exploit unique biological characteristics of hepatic tumours are entering clinical trial.


Br. J. Cancer (1994), 69, 372–378 © Macmillan Press Ltd., 1994

### Hepatic arterial chemotherapy for metastatic colorectal carcinoma

P.G. de Takats[1], D.J. Kerr[1], C.J. Poole[1], H.W. Warren[3] & C.S. McArdle[2]

[1]*Department of Clinical Oncology, Queen Elizabeth Hospital, Birmingham B15 2TH, UK;* [2]*University Department of Surgery, Royal Infirmary, Glasgow G31 2ER, UK.*

**Summary** In this review, the rationale of regional chemotherapy for treatment of hepatic metastases in advanced colorectal carcinoma is discussed. Pharmacokinetic principles and early clinical experience of hepatic arterial drug administration are summarised. The regional advantage of fluoropyrimidine compounds in this setting is well established, and recent evidence suggests that 5-fluorouracil (5-FU) is more efficacious than the analogue 5-fluoro-2′-deoxyuridine (FUDR). However, while significantly higher clinical response rates can be achieved with hepatic arterial infusion (HAI) chemotherapy compared with conventional intravenous drug administration, patient survival benefit is not significantly different. Several novel approaches to overcome the limitations of HAI therapy are currently being explored. These include concomitant use of biodegradable microspheres, which both slow tumour blood flow and enhance tumour drug uptake, and use of vasoactive agents to redistribute arterial blood flow towards tumours. In addition, novel chemotherapeutic agents which exploit unique biological characteristics of hepatic tumours are entering clinical trial.

The conventional notion of chemotherapy is of a truly systemic treatment designed to combat widely disseminated malignant disease. Colorectal carcinoma is relatively unresponsive to chemotherapy (Mayer, 1992), despite an increasing understanding of drug mechanisms of action at the molecular level. To date, systemic 5-FU plus folinic acid is considered the optimum treatment for metastatic colorectal cancer, yielding response rates of around 25% and median survival of around 12 months (Piedbois *et al.*, 1992). These disappointing results reflect, in part, the narrow therapeutic ratio of 5-FU and the presence of associated severe systemic side-effects limiting dose escalation. Regional chemotherapy affords an alternative method of cytotoxic drug administration which circumvents the constraints of systemic administration, while introducing the concept of targeted drug delivery to metastatic disease localised to specific components of the body.

Three levels of tumour targeting have been described (Widder *et al.*, 1979): (1) selective drug delivery to the tumour-bearing organ, (2) drug delivery biased to tumour rather than to normal tissue within that organ and (3) enhancement of uptake of cytotoxic drug by malignant cells. In this context, regional chemotherapy constitutes first-order targeting. Currently, the value of this method of drug delivery is being assessed in a number of different malignancies, but experience is greatest in the treatment of hepatic metastases from colorectal carcinoma.

Colorectal carcinoma is the second most common malignancy in the UK and the cause of over 19,000 deaths per year. Epidemiological studies show that the incidence of colorectal carcinoma is increasing, with approximately 28,000 new cases now being diagnosed annually. Up to 50% of patients with colorectal carcinoma develop liver metastases, from which as many as 70% of patients will subsequently die (Finlay & McArdle, 1986). As a consequence of the portal venous drainage system, colorectal cancers metastasise early to the liver, and post-mortem findings indicate that hepatic metastases may be the only site of disease spread in 20–30% of patients (Welch & Donaldson, 1979). The presence of hepatic metastases is a major prognostic indicator, survival being largely determined by the extent of hepatic disease at clinical presentation (Kemeny *et al.*, 1989*a*).

The median survival of patients with multiple metastases is 3-5 months in most series (Fortner *et al.*, 1984). For patients with isolated liver metastases, surgical resection may be the best treatment option, with 20–30% 5-year survival rates reported in non-randomised trial settings (Wagner *et al.*, 1984; Hughes *et al.*, 1986). However, such surgery is only possible in about 10% of patients and palliative chemotherapy is currently offered to most patients with extensive or multiple liver metastases. In view of the poor results obtained with systemic chemotherapy in this context, the potential to improve both patient response and survival by directly targeting treatment to the liver would appear, intellectually, highly attractive.

*Pharmacological rationale for regional chemotherapy*

The rationale for regional drug therapy in the management of hepatic metastases is the finding that established malignant lesions (greater than 1 cm diameter) derive most of their blood supply from the hepatic artery, in contrast to normal hepatocytes, which have a dual supply via the portal venous circulation (Stagg *et al.*, 1984). The concept of delivering chemotherapy to the liver via hepatic arterial infusion dates as far back as 1959 (Sullivan *et al.*, 1959), and over the following 20 years the pharmacological principles governing regional drug delivery were established.

The principal advantage of regional chemotherapy over conventional systemic therapy is the possibility of achieving higher drug concentrations at the tumour site while reducing systemic exposure and hence toxicity (Dedrick *et al.*, 1978). The major determinant for such a therapeutic advantage is the ratio of total body clearance to regional exchange rate, and mathematical formulae for pharmacokinetic models have been determined (Chen & Gross, 1980; Collins, 1984). The pharmacokinetic advantage of intra-arterial drug administration ($R_d$) can be quantitatively expressed by the following equation.

$$R_d = 1 + \frac{Cl_{TB}}{Q\ (1 - E)}$$

where $Cl_{TB}$ is the total body clearance obtained during intravenous infusion, $Q$ is the blood flow through the treated organ and $E$ is the extraction ratio (the fraction of drug that is extracted during a single pass through the treated organ). The ideal drug for hepatic arterial infusion would have a high total body clearance and a high extraction rate by the target organ (Table I). In addition, the slower the blood flow through the target organ, the greater the extraction ratio and the greater the regional advantage. Thus, hepatic arterial infusion of the anti-metabolite 5-FU or its analogue, FUDR, would theoretically maximise drug exposure to the tumour capillary bed, but result in low systemic levels and minimal subsequent toxicity to the patient (Ensminger *et al.*, 1978).

Correspondence: D.J. Kerr.
Received 7 July 1993.

**Table I** Pharmacokinetic characteristics of drugs commonly used for hepatic arterial administration

| *Drug* | *Estimated increase in hepatic exposure by HAI over peripheral infusion* | *Hepatic extraction ratio* |
|---|---|---|
| FUDR | × 100–400 | 0.95 |
| 5-FU | × 50–100 | 0.30–0.40 |
| Doxorubicin | × 1–10 | 0.45–0.50 |
| Mitomycin C | × 3–5 | 0.10–0.20 |

### *Clinical experience with hepatic arterial chemotherapy*

Since the introduction of intra-arterial infusion of chemotherapy to the liver by Sullivan and colleagues, early reports of treating patients with hepatic metastatic colorectal cancer claimed objective response rates in excess of 50%, associated with an increase in survival compared with historical control groups (Huberman, 1983). However, arterial placement of exteriorised rigid Teflon catheters in these uncontrolled trials by either radiographic or surgical means was associated with frequent patient morbidity, related to catheter displacement (1–75% prevalence), arterial or catheter thrombosis (1–22% prevalence), gastroduodenal haemorrhage (0–9% prevalence) and sepsis. The only prospective randomised controlled trial performed at this time comparing HAI with systemic therapy was reported by the Central Oncology Group (Grage *et al.*, 1979), in which 61 patients were randomised to receive either 21 days of a regional infusion with 5-FU or systemic bolus 5-FU. No significant difference in response rates or survival duration between the two groups was observed (Table II). However, the intra-arterial treatment was associated with a greater incidence of nausea, vomiting and diarrhoea, in addition to technical complications related to catheter insertion. Subsequent improvements in catheter technology and infusion pump design and surgical placement techniques have both simplified and facilitated a previously hazardous method of drug delivery, with consequent reduction in patient morbidity (Ensminger, 1987; Niederhuber & Grochow, 1989).

The presumed regional advantage of the 5-FU analogue FUDR, by virtue of its greater hepatic extraction, has been tested in five randomised trials for advanced colorectal carcinoma with liver metastases, comparing 2 weeks' continuous HAI of FUDR every 4 weeks against systemic therapy (Table II). These studies all made use of the more recently developed implantable constant-infusion device, enabling longer, more tolerable infusions than was previously possible by means of an external pump. Consequently, it is now generally accepted that statistically significantly higher response rates can be achieved with regional therapy (42–62%) than systemic therapy (10–21%). However, only a marginal survival advantage is apparent. The only one of these trials to claim a statistically significant improvement in survival is the French Consortium study, but it should be noted that half the patients in the systemic arm of this trial received no chemotherapy whatsoever.

It is argued that the lack of ability of these trials to demonstrate significant survival benefit is in part due to some of the studies having a cross-over facility between intravenous and intra-arterial groups in case of treatment failure. However, in these and previous non-randomised regional chemotherapy studies, between 40 and 80% of patients treated with regional chemotherapy for solitary hepatic metastases develop extrahepatic recurrence from which most patients ultimately die (Niederhuber *et al.*, 1984). In addition, of particular concern is the unacceptably high degree of local toxicity associated with regional FUDR. Biliary sclerosis occurred in 50% of patients in the French study and chemical hepatitis was documented in 42–79% of patients overall. Other frequent complications included gastric ulceration and cholecystitis. Significant technical complications were apparent, including catheter displacement, catheter thrombosis, hepatic artery thrombosis, pump pocket haematoma and peritonitis.

The disappointing results of regional chemotherapy demonstrated by these randomised trials has, not surprisingly, led to considerable controversy concerning the value of this approach to the treatment of patients with hepatic metastases (Kemeny, 1992; O'Connell, 1992). However, despite such misgivings, these trials clearly demonstrate that regionally administered chemotherapy can modify the progression of liver metastases and provide a striking change in the natural history of the disease, in that most patients die from extrahepatic metastatic recurrence. In addition, significantly higher quantities of cytotoxic agent can be administered while avoiding the usual side-effects associated with systemic therapy. Thus, in the absence of any new drugs with potential to treat metastatic colorectal carcinoma, the challenge to improve upon these trial results by manipulating this novel drug delivery system continues to stimulate scientists and clinicians alike.

### *New perspectives with 5-FU*

A significant limitation of HAI chemotherapy is its failure to prevent recurrence of disease outside of the liver, an event which ultimately leads to patient death. Two alternative strategies have been advanced to address this problem, aimed at achieving high therapeutic drug levels both within the liver and in the systemic circulation. Firstly, regional and systemic chemotherapy could be combined. Secondly, high-dose chemotherapy could be administered with the intention to cause overspill into the systemic circulation.

A number of phase II trials have combined hepatic arterial infusion of FUDR with mitomycin C. By virtue of low hepatic extraction on hepatic arterial administration, the advantage of therapeutic systemic levels of mitomycin C can

**Table II** Summary of randomised trials with intrahepatic *vs* systemic chemotherapy

| | *No. of patients* | *Trial drugs rate (%)* HAI | Systemic | *Hepatic response time (months)* HAI | Systemic | *Median survival* HAI | Systemic |
|---|---|---|---|---|---|---|---|
| COG | 61 | 5-FU | 5-FU | 34 | 23 | 10 | 13 |
| MSKCC[a] | 99 | FUDR | FUDR | 50 | 20 | 17 | 12 |
| NCOG[a] | 115 | FUDR | FUDR | 42 | 10 | 17 | 16 |
| NCI | 50 | FUDR | FUDR | 62 | 17 | 17 | 11 |
| NCCTG | 55 | FUDR | 5-FU | 54 | 21 | 13 | 11 |
| French Consortium | 163 | FUDR | 5-FU | 43 | 9 | 15 | 11 |

[a]Cross-over design. Abbreviations: COG, Central Oncology Group, (Grage *et al.*, 1979); MSKCC, Memorial Sloan Kettering Cancer Centre (Kemeny *et al.*, 1987); NCOG, Northern California Oncology Group (Hohn *et al.*, 1989); NCI, National Cancer Institute (Chang *et al.*, 1987); NCCTG, National Cancer Chemotherapy Group (Martin *et al.*, 1990).
French Consortium, Rougier *et al.* (1992).

be achieved. However, co-administration of these drugs has been prematurely abandoned because of an unacceptably high frequency of arterial thrombosis. Safi *et al.* (1989) reported the results of a phase III study using implanted pumps with dual outflow catheters, enabling both hepatic arterial and intravenous delivery of FUDR. Extrahepatic recurrence was reduced from 61% in 23 patients treated with HAI FUDR alone to 33% in 21 others treated with combined hepatic arterial and intravenous FUDR. Disappointingly, survival for the two groups was the same. However, a highly significant difference in survival time was evident between patients who responded to treatment and those whose liver metastases remained stable or progressed (median survival time 31 months *vs* 16 months), thus demonstrating the potential benefit of regional chemotherapy in certain patients.

A number of studies have shown that the elimination kinetics of 5-FU is non-linear, with both systemic clearance and hepatic extraction of the drug decreasing at very high dose rates (Wagner *et al.*, 1986). These observations are consistent with the loss in selective regional advantage achieved with hepatic arterial infusion of 5-FU administered at the maximum tolerated dose. What initially appeared to be a negative feature of regional 5-FU administration has subsequently been recognised to be a positive advantage for achieving both intrahepatic and extrahepatic disease control. While the solubility and potency of 5-FU is lower than that of FUDR, necessitating higher volume infusions, an external pump and surgical or radiological placement of arterial catheter, regional 5-FU is considerably less toxic than FUDR and hepatobiliary toxicity is not a feature.

In a series of pharmacokinetically guided studies, it has been shown that 24 h hepatic arterial infusion of 5-FU confers significant pharmacological advantage relative to intravenous infusions or intra-arterial bolus administration (Goldberg *et al.*, 1988, 1990). Further evidence suggests that modulation of regional 5-FU administration with folinic acid might confer significant therapeutic advantage (Anderson *et al.*, 1991). Unfortunately, regional administration of folinic acid precipitated hepatic artery occlusion in some patients, while in previous studies with regional FUDR (Kemeny *et al.*, 1989*b*), it had been shown to potentiate the risk of biliary sclerosis. Therefore, folinic acid is now administered systemically only.

A recent phase I study was performed with the aim of generating high intrahepatic 5-FU concentrations while maintaining adequate therapeutic systemic levels. The recommended dose of hepatic arterial 5-FU as a 24 h infusion when given in combination with a fixed dose of intravenous folinic acid ($400\ mg\ m^{-2}$) once per week (Anderson *et al.*, 1992) was found to be $1.5\ g\ m^{-2}\ week^{-1}$. At this dosage, neither myelosuppression nor hepatoxicity was apparent, while dose escalation to $2.0\ g\ m^{-2}\ week^{-1}$ 5-FU was associated with WHO grade 3/4 diarrhoea and vomiting. Pharmacokinetic data comparing intrahepatic and intravenous 5-FU indicated that the maximum tolerated dose of 5-FU was related to systemic 5-FU exposure, implying that there was overspill of drug into the circulation (J.H. Anderson, personal communication).

Based upon these data, a phase II study was performed. Thirty patients with histologically proven metastases confined to the liver received weekly 24 h infusion of 5-FU ($1.5\ g\ m^{-2}\ week^{-1}$) via an indwelling hepatic arterial catheter, with folinic acid ($400\ mg\ m^{-2}$) administered intravenously. The response rate of 27 evaluable patients was 44%, with median survival 18 months. These data compare favourably with those of previous HAI phase III trials. Of particular significance following the FUDR experience is the minimal toxicity associated with HAI of 5-FU. The therapeutic potential for this regional drug combination is to be tested in an MRC-sponsored phase III clinical trial, randomising pharmacokinetically equivalent intravenous and HAI 5-FU plus folinic acid regimens. The conclusions from this trial will be a major determinant of subsequent continued research interest in HAI chemotherapy in the UK.

### *Biodegradable microspheres*

Biodegradable starch microspheres, approximately 40 μm in diameter, when injected into the hepatic artery, have been shown to lodge in the microvasculature and block flow for 15–30 min (Dakhil *et al.*, 1982). Co-administration of microspheres and cytotoxic drug results in the drug being trapped in a relatively stationary fluid column, allowing greater exposure time to the surrounding tissues. In an early report, a bolus injection of mitomycin with $9 \times 10^7$ microspheres resulted in up to 70% reduction in systemic drug exposure (Gyves *et al.*, 1983) with 4- to 9-fold increase in hepatic extraction compared with intravenous administration of mitomycin C (MMC). Such potential therapeutic advantage could not be reproduced by Goldberg *et al.* (1991*a*), who found that arteriovenous shunting in seven patients with colorectal metastases was minimal (2.2 ± 1.8%) and was not significantly increased by regional administration of a therapeutic quantity – $0.5 \times 10^6$ – of microspheres (3.0 ± 0.8%). Indeed, in a controlled trial of 61 similar patients (Hunt *et al.*, 1990) randomised to receive either hepatic artery embolisation, HAI with 5-FU and microspheres or no active intervention, median survival of treated patients *vs* controls (9 months, 13 months and 10 months respectively) was not significantly different. However, patients with low-volume hepatic disease appeared to fare better.

An alternative approach has been to encapsulate drugs within injectable particles. MMC (Kato *et al.*, 1980) has been incorporated into ethylcellulose microcapsules, but these microcapsules are not biodegradable and permanently occlude the vessels in which they lodge. Although resulting tumour infarction and ensuing hypoxia enhance the therapeutic effect of prolonged regional drug exposure of MMC, this effect precludes repeated courses of treatment, while also damaging normal hepatic parenchyma. Attempts have been made to microencapsulate MMC using polylactide-glycolide polymers, and initial formulations have produced a 20–30 μm microcapsule with appropriate drug-release characteristics. A phase I clinical trial has been initiated (Whateley *et al.*, 1992) and it is conceivable, given the non-overlapping toxicities of MMC and 5-FU, that the microcapsules serve an additional role in current strategies of HAI of 5-FU in hepatic metastatic colorectal carcinoma.

### *Use of vasoactive agents to modify tumour blood flow*

The distribution of arterially administered chemotherapy reflects the pattern of arterial blood flow within the liver. The disappointing results of regional chemotherapy may be due to the relatively hypovascular nature of hepatic metastases (Taylor *et al.*, 1979), limiting homogeneous drug delivery to the tumour. Indeed the presence of hypovascularised metastases, as defined by radionuclide liver scan with technetium-99m-labelled microaggregated albumin, is recognised as a negative prognostic determinant of response in patients receiving hepatic arterial chemotherapy (Rougier *et al.*, 1991).

Vasoactive agents have been used in animal models to modify arterial blood flow, by causing temporary arteriolar constriction in normal blood vessels (Burton *et al.*, 1985). Newly formed blood vessels in tumour tissue lack both smooth muscle and adrenergic innervation (Mattson *et al.*, 1977) and are therefore less responsive to vasoactive drugs than those of normal liver tissue. The vasoconstrictor angiotensin II has been shown to increase blood flow to hepatic metastases relative to normal tissue in patients (Sasaki *et al.*, 1985). A 4 min infusion of angiotensin II ($10\ \mu g\ min^{-1}$) via a hepatic artery catheter induced an increase in tumour blood flow by approximately 300% relative to the normal liver. By the same mechanism, angiotensin II could potentially influence drug targeting. This was shown by Goldberg *et al.* (1991*b*), who performed scintigraphic studies of the liver in nine patients with colorectal liver metastases after (1) intravenous injection of albumin colloid, (2) hepatic arterial injection of a tracer amount of radiolabelled albumin

**Table III** Pharmacokinetic studies of intravenous (i.v.) and HAI 5-FU with and without albumin microspheres and angiotensin II in patients with advanced colorectal liver metastases

| *Treatment regimen* | *No. of patients* | *5-FU regimen* | *AUC (mg l min⁻¹)* | *Clearance (l min⁻¹)* | $t_{½}$ *(min)* |
|---|---|---|---|---|---|
| 5-FU | 9 | i.v. 1 g bolus | 1172 ± 365 | 0.94 ± 0.3 | 17 ± 5 |
| 5-FU | 7 | i.v. 1 g 2 h infusion | 1200 ± 262 | 1.7 ± 0.2 | – |
| 5-FU | 5 | i.v. 1 g 24 h infusion | 54 ± 18 | 18 ± 7 | – |
| 5-FU | 9 | HAI 1 g bolus | 1312 ± 325 | 0.81 ± 0.2 | 17 ± 6 |
| AMS + 5-FU | 9 | HAI 1 g bolus | 1115 ± 481 | 1.01 ± 0.3 | 17 ± 6 |
| AII + AMS + 5-FU | 9 | HAI 1 g bolus | 1403 ± 461 | 0.78 ± 0.3 | 7 ± 3 |
| 5-FU | 7 | HAI 1 g 2 h infusion | 788 ± 104 | 2.7 ± 0.4 | – |
| 5-FU | 5 | HAI 1 g 24 h infusion | 24 ± 18 | 42 ± 27 | – |

Abbreviations: AUC, area under the curve; $t_{½}$, plasma half-life of 5-FU; AMS, albumin microspheres, 350 mg; AII, angiotensin II, 10 μg min⁻¹.

Values expressed as mean ± standard deviation. All 5-FU concentrations were measured in peripheral venous plasma.

microspheres and (3) hepatic arterial injection of albumin microspheres given immediately after a 100 s arterial infusion of 10 μg of angiotensin II. The median tumour–normal ratio of radioactivity determined by both scintigraphic planar and tomographic imaging was 3.4:1 before and 7.3:1 after angiotensin II administration. In a further study, scintillation counting of paired tumour and normal liver biopsies taken from patients following angiotensin II and radiolabelled microsphere tracer administration showed that the uptake of microspheres in tumour was 3-fold greater than that in normal liver (Goldberg *et al.*, 1991*c*). Tested in phase II clinical trial (Goldberg *et al.*, 1990), 21 patients receiving an infusion of angiotensin II (10 μg min⁻¹) followed by bolus HAI of microspheres and 1 g at 5-FU 4 to 6 weekly tolerated treatment well. Seven patients showed clinical response, while the median survival of 9 months suggested some significant therapeutic benefit compared with that of historical controls.

Unfortunately, the effect of angiotensin II is short-lived, while prolonged infusion causes significant rises in systemic blood pressure. Alternative vasoactive agents including vasopressin (Jenkins *et al.*, 1984) and verapamil (Kaelin *et al.*, 1982) have shown therapeutic potential in animal tumour models, but their clinical application remains to be explored. In clinical practice, use of angiotensin II with or without albumin microspheres has not been shown to significantly alter the pharmacokinetics of bolus HAI 5-FU (Table III). It would appear that current optimum regional chemotherapy with 5-FU involves prolonged infusion, and enhancement of 5-FU cytotoxicity by any chemico-biological agent other than folinic acid remains to be proven.

### *Arterial administration of macromolecules*

Most conventional chemotherapeutic agents are small molecules of less than 2 kDa in size, with short half-lives as a consequence of rapid renal excretion. Macromolecules of 50 kDa or more in size have been shown to accumulate passively within tumours as a result of increased vascular permeability of tumours and their lack of a lymphatic drainage system (Maeda *et al.*, 1992). SMANCS (Maeda *et al.*, 1984) is a 15.5 kDa conjugate of the anti-tumour protein neocarzinostatin (NCS) and two chains of the synthetic co-polymer styrene–maleic acid (SMA), which binds to albumin in plasma, with an effective molecular weight of around 83 kDa (Figure 1). Polymer conjugation of NCS affords a 10-fold increase in half-life and enhanced stability, both *in vitro* and *in vivo*.

The Japanese have accumulated over 10 years of experience with SMANCS, which has now been administered to

**Figure 1** **a**, Structure of SMA, poly(styrene-co-maleic acid/anhydride) half-butylester. **b**, Diagrammatic representation of the reaction with NCS to produce the conjugate SMANCS.

over 200 patients with primary hepatocellular carcinoma. HAI of SMANCS administered as a formulation with the lipid contrast medium Lipiodol is associated with a tumour–systemic drug concentration ratio of greater than 2,500. In a pilot phase II study (Konno *et al.*, 1983), 44 patients with advanced hepatoma received a total of 88 bolus injections of SMANCS/Lipiodol via the hepatic artery. Treatment resulted in a decrease in alpha-fetoprotein in 86% of patients and a reduction in tumour size in 95% of patients. While the median survival of such patients in Japan treated conventionally with chemotherapy plus or minus surgery is around 6 months, there is now accumulating data which show that survival of patients 3 years after treatment with, on average, four courses of HAI SMANCS/Lipiodol ranges between 30 and 90%, depending on the extent of liver involvement and the presence or absence of cirrhosis (Maeda, 1991).

Furthermore, it would appear that SMANCS has broad-spectrum activity, being effective against a number of different solid tumours. Konno *et al.* (1984) reported their experience of treating 24 patients with a variety of solid tumours, 11 of whom had colorectal hepatic metastases. HAI with SMANCS/Lipiodol resulted in tumour response in ten of these patients, four of whom underwent subsequent resection of their residual tumour. The median survival of patients with unresectable tumours was 8 months, compared with around 4 months for untreated patients. Associated toxicity is remarkably low, comprising low-grade fever and mild abdominal discomfort only, while biochemical and haematological changes comprise only a transient rise in liver enzymes and moderate leucocytosis. It is anticipated that SMANCS will shortly be entering clinical trial in association with the Liver Unit, Queen Elizabeth Hospital, Birmingham, UK.

### *Patient selection in future HAI trials*

There is now sufficient clinical trial data available on regional chemotherapy to enable the definition of clinical parameters which may predict both response to treatment and survival, in order to select patients most likely to benefit from regional chemotherapy (Kemeny *et al.*, 1989*a*; Rougier *et al.*, 1991). The most important factor affecting survival appears to be the extent of liver involvement, while the only significant factor predicting response to hepatic arterial chemotherapy is perfusion character: hypovascular tumours respond poorly compared with well-perfused tumours. Methods for detecting occult hepatic metastases in colorectal cancer which are being developed include duplex sonography (Leen *et al.*, 1991) and dynamic hepatic scintigraphy (Hemingway *et al.*, 1992). Earlier detection leading to more rapid referral of patients with low-volume hepatic metastatic disease should result in improved response to regional chemotherapy and ultimate gain in patient survival.

### Conclusions

Systemic chemotherapy administration in metastatic colorectal carcinoma is limited by the inherent resistance of this tumour type to conventional cytotoxic drugs and by the steep dose–response curve of conventional drugs, since dose escalation is hindered by unacceptable toxicity. Regional drug delivery to the major site of tumour burden has been extensively researched in the treatment of colorectal hepatic metastases. Initial optimism based on sound mathematical and pharmacokinetic principles has, over the years, been dampened by the inability to demonstrate theoretical potential in clinical practice. However, more recent innovative strategies aimed at exploiting inherent biological and pharmacological characteristics have revitalised this field of research, enabling new avenues to be explored.

Clearly, taking into account the high costs incurred in administering regional chemotherapy, both to the patient and financially, regional chemotherapy will remain an investigational procedure until significant therapeutic advantage in terms of survival benefit can be demonstrated. However, the desperate predicament of large numbers of patients for whom there is currently little option for treatment provides a stimulus to all cancer physicians and surgeons to maintain an interest in hepatic arterial drug delivery systems and support randomised trials in this area.

### References

ANDERSON, J.H., KERR, D.J., SETONIANS, A., COOKE, T.G. & MCARDLE, C.S. (1991). A pharmacokinetic comparison of intravenous versus intra-arterial folinic acid. *Br. J. Cancer*, **65**, 133–135.

ANDERSON, J.H., KERR, D.J., COOKE, T.G. & MCARDLE, C.S. (1992). A phase I study of regional 5-fluoruracil and systemic folinic acid for patients with colorectal liver metastases. *Br. J. Cancer*, **65**, 913–915.

BURTON, M.A., GRAY, B.N., HEGGIE, J. & TOWNSEND, P.S. (1985). Manipulation of experimental rat and rabbit tumour blood flow with angitensin II. *Cancer Res.*, **45**, 5390–5393.

CHANG, A.E., SCHNEIDER, P.D., SUGARBAKER, P.H., SIMPSON, C., CULNANE, & STEINBERG, S.M. (1987). A prospective randomized trial of regional versus systemic continuous 5-fluordeoxyuridine chemotherapy in the treatment of colorectal liver metastases. *Ann. Surg.*, **206**, 685–693.

CHEN, H.G. & GROSS, J.F. (1980). Intra-arterial infusion of anticancer drugs: theoretic aspects of drug delivery and review of responses. *Cancer Treat. Rep.*, **64**, 31–40.

COLLINS, J.M. (1984). Pharmacologic rationale for regional drug delivery. *J. Clin. Oncol.*, **2**, 498–504.

DAKHIL, S., ENSMINGER, W.D., CHO, K., NIEDERHUBER, J., DOAN, K. & WHEELER, R. (1982). Improved regional selectivity of hepatic arterial BCNU with degradable microspheres. *Cancer*, **50**, 631–635.

DEDRICK, R.L., MYERS, C.E., BUNGAY, P.M. & DEVITA, V.T. (1978). Pharmacokinetic rationale for peritoneal drug administration in the treatment of ovarian carcinoma. *Cancer Treat. Rep.*, **62**, 1–11.

ENSMINGER, W.D. (1987). Intrarterial chemotherapy for the treatment of hepatic metastases. In *Principles and Practice of Oncology*, Devita, Jr, V.T., Hellman, H.S. & Rosenberg, S.A. (eds), pp. 1–11. Lippincott: Philadelphia.

ENSMINGER, W.D., ROSOWSKI, A., RASO, V., LEVIN, D.C., GLODE, M., COME, S., STEELE, G. & FREI, E. (1978). A clinical-pharmacological evaluation of hepatic arterial infusions of 5-fluor-2′-deoxyuridine and 5-fluorouracil. *Cancer Res.*, **38**, 3784–3792.

FINLAY, I.G. & MCARDLE, C.S. (1986). Occult hepatic metastases in colorectal carcinoma. *Br. J. Surg.*, **73**, 732–735.

FORTNER, J.J., SILVA, J.S., COX, E.B., GOLBY, R.B., GALLOWITZ, H. & MACLEAN, B.J. (1984). Multivariate analysis of a personal series of 247 patients with liver metastases from colorectal cancer. *Ann. Surg.*, **19**, 306–316.

GOLDBERG, J.A., KERR, D.J., WILMOTT, N., MCKILLOP, J.H. & MCARDLE, C.S. (1988). Pharmacokinetics and pharmacodynamics of locoregional 5-fluorouracil (5FU) in advanced colorectal liver metastases. *Br. J. Cancer*, **57**, 186–189.

GOLDBERG, J.A., KERR, D.J., WILMOTT, N., MCKILLOP, J.H. & MCARDLE, C.S. (1990). Regional chemotherapy for colorectal metastases: a phase II evaluation of targeted hepatic arterial 5-fluorouracil for colorectal liver metastases. *Br. J. Surg.*, **77**, 1238–1240.

GOLDBERG, J.A., THOMSON, J.A.K., MCCURRACH, G., ANDERSON, J.H., WILMOTT, N., BESSENT, R.G., MCKILLOP, J.H. & MCARDLE, C.S. (1991*a*). Arteriovenous shunting in patients with colorectal liver metastases. *Br. J. Cancer*, **63**, 466–468.

GOLDBERG, J.A., THOMSON, J.A.K., BRADNAM, M.S., FENNER, J., BESSENT, G., MCKILLOP, J.H., KERR, D.J. & MCARDLE, C.S. (1991*b*). Angiotensin II as a potential method of targeting cytotoxic-loaded microspheres in patients with colorectal liver metastases. *Br. J. Cancer*, **64**, 114–119.

GOLDBERG, J.A., MURRAY, T., KERR, D.J., WILMOTT, N., BESSENT, R.G., MCKILLOP, J.H. & MCARDLE, C.S. (1991*c*). The use of angiotensin II as a potential method of targeting cytotoxic microspheres in patients with intrahepatic tumour. *Br. J. Cancer*, **63**, 308-310.

GRAGE, T.B., VASSILOPOULOS, P.P., SHINGLETON, W.W., JUBERT, A.V., ELIAS, E.G., AUST, J.B. & MOSS, S.E. (1979). Results of a prospective randomized study of hepatic artery infusion with 5-fluoruracil versus intravenous 5-fluoruracil in patients with hepatic metastases from colorectal cancer: A Central Oncology Group study. *Surgery*, **86**, 550-555.

GYVES, J.W., ENSMINGER, W.D., VANHARKEN, D., NIEDERHUBER, J.E., STETSON, P. & WALKER, S. (1983). Improved regional selectivity of hepatic arterial mitomycin by starch microspheres. *Clin. Pharmacol. Ther.*, **34**, 259-265.

HEMINGWAY, D.M., COOKE, T.G., MCCURRACH, G., BESSENT, R.G., CARTER, J.H., MCKILLOP, J.H. & MCARDLE, C.S. (1992). Clinical correlation of high activity dynamic hepatic scintigraphy in patients with colorectal cancer. *Br. J. Cancer*, **65**, 781-782.

HOHN, D.C., STAGG, R.J., FRIEDMAN, M.A., HANNIGAN, J.F. Jr, RAYNER, A., IGNOFFO, R.J., ACORD, P. & LEWIS, B.J. (1989). A randomized trial of continuous intravenous versus hepatic intra-arterial floxuridine in patients with colorectal cancer meatstatic to the liver. *J. Clin. Oncol.*, **7**, 1646-1654.

HUBERMAN, M.S. (1983). Comparison of systemic chemotherapy with hepatic arterial infusion in metastatic colorectal carcinoma. *Semin. Oncol.*, **10**, 238-248.

HUGHES, K.S., SIMON, R., SONGHORABODI, S., ADSON, M.A., ILSTROP, D.M., FORTNER, J.G., MACLEAN, B.J., FOSTER, J.H. & DALY, J.M. (1986). Resection of liver for colorectal metastases: a multi-institutional study of patterns of recurrence. *Surgery*, **100**, 278-284.

HUNT, T.M., FLOWERDEW, A.D.S., BIRCH, S., WILLIAMS, J.D., MULLEE, M.A. & TAYLOR, I. (1990). Prospective randomized controlled trial of hepatic arterial embolization or infusion chemotherapy with 5-fluoruracil and degradable starch microspheres for colorectal liver metastases. *Br. J. Surg.*, **77**, 779-782.

JENKINS, S.A., DAY, D.W., MOONEY, B., DVITT, P., TAYLOR, I. & SHIELDS, R. (1984). The effect of vasopressin and hepatic artery ligation on the blood supply to normal and metastatic liver tissue. *Br. J. Cancer*, **50**, 785-791.

KAELIN, Jr, W.G., SHRIVASTAV, S., SHAND, D.G. & JIRTLE, R.L. (1982). Effect of verapamil on malignant tissue blood flow in SMT-2A tumour bearing rats. *Cancer Res.*, **42**, 3944-3949.

KATO, T., NEMOTO, R., MORI, H. & KUMAGAI, I. (1980). Sustained release properties of microencapsulated mitomycin C with ethycellulose infused into the renal artery of the dog kidney. *Cancer*, **46**, 14-21.

KEMENY, N. (1992). Is hepatic infusion of chemotherapy effective treatment for liver metastases? Yes! In *Important Advances in Oncology 1992*, DeVita, V.J., Hellman, S. & Rosenberg, S. (eds), pp. 207-227. Lippincott: Philadelphia.

KEMENY, N., DALY, J., REICHMANN, B., GELLER, N., BOTET, J. & ODERMAN, P. (1987). Intrahepatic or systemic infusion of fluorodeoxyuridine in patients with liver metastases from colorectal carcinoma. A randomized trial. *Ann. Intern. Med.*, **107**, 459-465.

KEMENY, N., NIEDZWIECKI, D., SHURGOT, B. & ODERMAN, P. (1989*a*). Prognostic variables in patients with hepatic metastases from colorectal cancer. Importance of medical assessment. *Cancer*, **63**, 742-747.

KEMENY, N., COHEN, A., BERTINO, J.R., SIGURDSON, E.R., BOTET, J. & ODERMAN, P. (1989*b*). Continuous intrahepatic infusion of floxuridine and leucovorin through an implantable pump for the treatment of hepatic metastases from colorectal carcinoma. *Cancer*, **65**, 2446-2450.

KONNO, T., MAEDA, H., IWAI, K., TASHIRO, S., MAKI, S., MORINAGA, T., MOCHINAGA, M., HIRAOKA, T. & YOKOYAMA, I. (1983). Effect of arterial administration of high-molecular-weight anticancer agent SMANCS with lipid lymphographic agent on hepatoma: a preliminary report. *Eur. J. Cancer Clin. Oncol.*, **19**, 1053-1065.

KONNO, T., MAEDA, H., IWAI, K., MAKI, S., TASHITO, S., USHIDA, M. & MIYAUCHI, Y. (1984). Selective targeting of anti-cancer drug and simultaneous image enhancement in solid tumors by arterially administered lipid contrast medium. *Cancer*, **54**, 2367-2374.

LEEN, E., GOLDBERG, J.A., ROBERTSON, J., SUTHERLAND, G.R. & MCARDLE, C.S. (1991). The use of duplex sonography in the detection of colorectal hepatic metastases. *Br. J. Cancer*, **63**, 323-325.

MAEDA, H. (1991). SMANCS and polymer-conjugated macromolecular drugs: advantages in cancer chemotherapy. *Adv. Drug Delivery Rev.*, **6**, 181-202.

MAEDA, H., MATSUMOTO, T., KONNO, T., IWAI, K. & UEDA, M. (1984). Tailor-making of protein drugs by polymer conjugation for tumor targeting. *J. Protein Chem.*, **3**, 181-193.

MAEDA, H., SEYMOUR, L.W. & MIYAMOTO, Y. (1992). Conjugates of anticancer agents and polymers: advantages of macromolecular therapeutics *in vivo*. *Bioconjugate Chem.*, **3**, 351-362.

MARTIN, J.K., O'CONNELL, M.J., WIEAND, H.S., FITZGIBBONS, R.J., MAILLIARD, J., NAGORNEY, D.M., TSCHETTER, L.K. & KROOK, Jr, J.E. (1990). Intra-arterial floxuridine *vs* systemic fluorouracil for hepatic metastases from colorectal cancer. *Arch. Surg.*, **125**, 1022-1027.

MATTSON, J., APPLEGREN, L., HAMBERGER, B. & PETERSON, H.I. (1977). Adrenergic innervation of tumour blood vessels. *Cancer Lett.*, **3**, 347.

MAYER, R.J. (1992). Chemotherapy for metastatic colorectal cancer. *Cancer*, **70** (Suppl.), 1414-1424.

NIEDERHUBER, J.E. & GROCHOW, L.B. (1989). Status of infusion chemotherapy for the treatment of liver metastases. In *Principles and Practice of Oncology*, Vol. 3, DeVita, V.J., Hellman, S. & Rosenberg, S. (eds), pp. 1-9. Lippincott: Philadelphia.

NIEDERHUBER, J., ENSMINGER, W., GYVES, J., THRALL, J., WALKER, S. & COZZI, E. (1984). Regional chemotherapy of colorectal cancer metastatic to the liver. *Cancer*, **53**, 1336-1343.

O'CONNELL, M.J. (1992). Is hepatic infusion of chemotherapy effective for liver metastases? No! In *Important Advances in Oncology 1992*, DeVita, V.J., Hellman, S. & Rosenberg, S. (eds), pp. 229-234. Lippincott: Philadelphia.

PIEDBOIS, P., BUYSE, M., RUSTUM, Y., MACHOVER, D., ERLICHMAN, C., CARLSON, R.W., VALONE, F., LABIANCA, R., DOROSHOW, J.H. & PETRELLI, N. (1992). Modulation of fluoruracil by leucovorin in patients with advanced colorectal cancer: evidence in terms of response rate. *J. Clin. Oncol.*, **10**, 896-903.

ROUGIER, P., DUCREUX, M., PIGNON, J.P., ELIAS, J.M., TIGAUD, J.M., LUMBROSO, J., RUFFIE, P. & LASSER, P.H. (1991). Prognostic factors in patients with liver metastases from colorectal carcinoma treated with discontinuous intra-arterial hepatic chemotherapy. *Eur. J. Cancer*, **27**, 1226-1230.

ROUGIER, P., LAPLANCHE, A., HUGUIER, M., HAY, J.M., OLLIVIER, J.M., ESCAT, J., SALMOM, R., JULIEN, M. & AUDY, J.R. (1992). Hepatic arterial infusion of floxuridine in patients with liver metastases from colorectal carcinoma: long-term results of a prospective randomized trial. *J. Clin. Oncol.*, **10**, 1112-1118.

SAFI, F., BITTNER, R., ROSCHER, R., SCHUMACHER, K., GAUS, W. & BEGER, G.H. (1989). Regional chemotherapy for hepatic metastases of colorectal carcinoma (continuous intraarterial versus continuous intraarterial/intravenous therapy). *Cancer*, **64**, 379-387.

SASAKI, Y., SHINGI, I., HASEGAWA, Y., NAKANO, S., ISHIKAWA, O., OHIYISHI, H., TANIGUCHI, K., KOYAMA, H. & IWANAGA, T. (1985). Changes in distribution of hepatic blood flow induced by intra-arterial infusion of angiotensin II in human hepatic cancer. *Cancer*, **55**, 311-316.

STAGG, R.J., LEWIS, B.J., FRIEDMAN, M.A., IGNOFFO, R.J. & HOHN, D.C. (1984). Hepatic arterial chemotherapy for colorectal cancer metastatic to liver. *Ann. Intern. Med.*, **100**, 736-743.

SULLIVAN, R.D., MILLER, E. & SYKES, M.P. (1959). A clinical-pharmacological evaluation of hepatic arterial infusions of 5-fluoro-2'-deoxyuridine and 5-fluoruracil. *Cancer Res.*, **38**, 3784-3792.

TAYLOR, I., BENNETT, R. & SHERRIF, S. (1979). The blood supply of colorectal liver metastases. *Br. J. Cancer*, **39**, 746-756.

WAGNER, J.G., GYVES, J.W., STETSON, P.L., WALKER-ANDREWS, S.C., WOLLNER, I.S., COCHRAN, M.K. & ENSMINGER, W.D. (1986). Steady-state nonlinear pharmacokinetics of 5-fluoruracil during hepatic arterial and intravenous infusions in cancer patients. *Cancer Res.*, **46**, 1499-2506.

WAGNER, J.S., ADSON, M.A., VAN HEERDEN, J.A., ADSON, M.H. & ILSTRUP, D.M. (1984). The natural history of hepatic metastases from colorectal cancer. A comparison with resective treatment. *Ann. Surg.*, **199**, 502–508.

WELCH, J.P. & DONALDSON, G.A. (1979). The clinical correlation of an autopsy study of recurrent colorectal cancer. *Ann. Surg.*, **189**, 496–502.

WHATELEY, T.L., ELEY, J.G., KERR, D.J., MCARDLE, C.S. & VERT, M. (1992). Poly(lactide-glucolide) microspheres containing mitomycin C. *Proceedings of the 8th International Symposium on Microencapsulation, Dublin*, pp 23–27.

WIDDER, K.J., SENYEI, A.E. & RANNEY, D.F. (1979). Magnetically responsive microspheres and other carriers for the biophysical targeting of antitumour agents. *Adv. Pharmacol. Chemother.*, **16**, 213–271.